# Treatment Effect of Zoledronic Acid in Chronic Non-bacterial Osteomyelitis of the Jaw: A Case Series

**DOI:** 10.1007/s00223-023-01154-4

**Published:** 2023-11-22

**Authors:** Rasmus Bo Jansen, Johanna Nilsson, Kristian Buch-Larsen, Thomas Kofod, Peter Schwarz

**Affiliations:** 1 Department of Endocrinology, Bone-Metabolic Research Unit, Rigshospitalet, Copenhagen, Denmark; 2 Department of Oral and Maxillofacial Surgery, Rigshospitalet Copenhagen, Denmark

**Keywords:** Osteomyelitis, Bone remodelling, Zoledronic acid, Chronic non-bacterial osteomyelitis of the jaw, Jaw pain

## Abstract

Chronic non-bacterial osteomyelitis (CNO) is an autoinflammatory, osteolytic bone disorder sometimes localized to a unifocal site in the jaw, causing long-term pain and reduced function. The aim of this study was to describe the patients with CNO of the jaw, focusing on treatment with zoledronic acid for pain relief. An analysis of medical records of 24 patients with CNO of the jaw, including treatment with zoledronic acid and effects on pain relief. Descriptive statistics and nonparametric tests were used to describe the population and compare treatment effects, respectively. The average treatment period was 33.4 months (median 23; Q1 11.5; Q3 42.0) with an average of 4.1 infusions (median 3; Q1 2; Q3 5) of zoledronic acid. The average pain VAS score (visual analogue scale) was significantly reduced from 7.7 (median 8; Q1 6.5; Q3 8.5) to 2.5 points (median 2; Q1 0.5; Q3 4.5) (p < 0.001). At final visit, 46% of patients reported no pain and 38% reported a reduction of pain. At least 67% of patients had at least one episode of pain recurrence, and most patients experienced the first recurrence within a year of initial treatment. Four patients (16%) had no pain relief from the treatment. In this group of patients with CNO of the jaw, there was a positive response to treatment with zoledronic acid on pain relief, averaging 5.2 points on a pain VAS score, with 84% of patients treated experiencing either a partial or a total reduction in pain after about 2.5 years.

**Supplementary Information:**

The online version contains supplementary material available at 10.1007/s00223-023-01154-4.

## Introduction

Chronic non-bacterial osteomyelitis (CNO) and chronic recurrent multifocal osteomyelitis (CRMO) are autoinflammatory, osteolytic disorders of the bone, affecting either one (CNO) or multiple sites (CRMO) [1–3].

The diagnosis of CNO/CRMO is difficult and often an exclusion diagnosis following negative evaluations for infection and malignancy. The diagnosis is commonly supported by a combination of the clinical presentation and radiological findings on either plain X-ray, Cone Beam Computer Tomography (CBCT), Magnetic Resonance Imaging (MRI) and/or Computer Tomography (CT) with or without Positron Emission Tomography (PET CT), sometimes aided by bone biopsies [4, 5].

Some patients with CNO only develop a single affected site related to the jaw area, most often unifocally in the posterior part of the mandible. The site of CNO activity can swell up, cause pain and limited mouth opening, and as such might be mistaken for a case of infectious osteomyelitis [6]. The management of CNO is challenging, and a wide range of treatment strategies have been described in the literature including long-term nonsteroidal anti-inflammatory drugs (NSAIDs), antibiotics, corticosteroids, methotrexate, bisphosphonates, as well as surgical decortication [7–12]. Patients are often initially assessed solely by dentists which might risk prolonging the already considerable diagnostic delay due to the difficulties of the diagnosis [13].

CNO is presenting at two incidence peaks. The first peak appears in children and young adolescents, also described as Garré's osteomyelitis. When jaw affection occurs in children, it is most often part of a multifocal CRMO presentation [14].

The second peak is observed in the age group > 50 years and is often appearing as an unifocal CNO of the jaw, as opposed to the multifocal CNO/CRMO affection seen in children and adolescents [6, 15]. As suggested by Buch et al., these unifocal CNO lesions of the jaw might form as a separate subgroup of CNO/CRMO cases (1).

In this study, we wanted to investigate the subgroup of CNO patients with unifocal lesions of the jaw. The aim was to describe a population of patients with CNO of the jaw, with a focus on the treatment effect of zoledronic acid with regard to pain relief.

## Methods

Patients were identified based on a referral diagnosis of CNO of the jaw from patients referred to the Department of Endocrinology at Rigshospitalet, Copenhagen, Denmark, in the period of 2008–2023. After identification, the patients’ electronic records were reviewed individually. Primary data collection were done retrospectively.

In addition, a short follow-up phone interview was conducted with each patient in February–March 2023, focusing on pain relief after treatment with intravenous zoledronic acid, as well as any subsequent symptoms of discomfort after end of treatment.

Data collected included anthropomorphic data, duration of symptoms prior to referral, duration of antibiotic treatment prior to referral, diagnostic imaging used to confirm the diagnosis of CNO, average number of infusions with zoledronic acid, treatment time, and pain relief during treatment including recurrence of pain after initial treatment.

All time points have been rounded to the nearest half month.

1. Duration of symptoms was defined as the time from first contact with the health care system (not including private dentists) to first visit to the outpatient clinic.
2. Duration of antibiotic treatment prior to referral was defined as months of documented continual use of antibiotics with no other indicated diagnosis than CNO. Some patients had other issues that required antibiotics within the same timeframe, and these treatments have been removed from the data.
3. The average treatment time was defined as the time from first infusion of zoledronic acid to final discharge or the date of March 1, 2023, whichever came first.
4. The main outcome recorded was pain relief after treatment.
  For pain relief, we used a simplified scale of “Unchanged pain,” “Reduced pain,” or “No pain” based on data in the medical records. For the interview, we used a Visual Analog Scale (VAS) of pain from 0 to 10, with 10 being the worst pain imaginable.
5. Recurrence of pain was defined as an increase in pain from the lowest level achieved after treatment with zoledronic acid. Thus, a recurrence did not necessarily mean that the patient had a pain increase corresponding to their initial level prior to treatment. No consistent clinical tests were made for verification with blood tests or imaging.

Treatment was done with one or more intravenous infusions of 4 mg zoledronic acid in 100 mL isotonic saline solution, infused over 30 min.

**Fig. 1:**
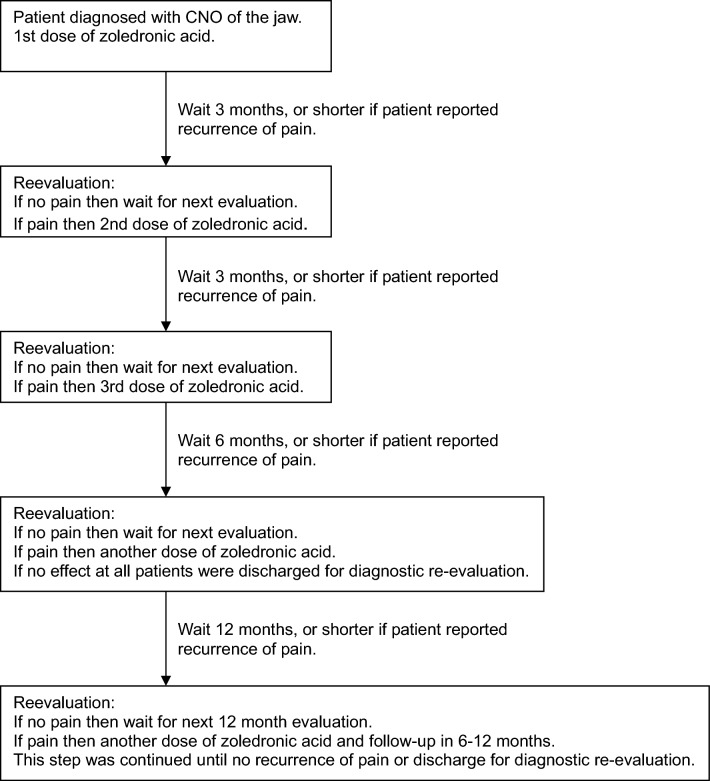
Flowchart of treatment regime with zoledronic acid for pain related to CNO of the jaw

All patients were given one dose of zoledronic acid at the first visit. The need for more than one dose of zoledronic acid was in general assessed using the flowchart in Fig. 1. Due to the heterogeneous nature of the CNO-related pain, and the retrospective nature of this study, some of the re-evaluations did not adhere strictly to the time table in the flowchart.

### Biochemistry

Blood samples were obtained by venipuncture in the antecubital vein and processed at the central laboratory at Rigshospitalet, Denmark. Blood samples were performed to ensure normal kidney function before treatment, and markers of bone turnover were measured to detect treatment effect of zoledronic acid by monitoring a lower level of bone resorption markers.

The analysis included plasma (p)-25-hydroxyvitamin D (p-25OHD), p-creatinine, p-alkaline phosphatase, p-albumin, p-ionized calcium (p-Ca^2+^), p-parathyroid hormone (p-PTH), p-bone-specific alkaline phosphatase, p-osteocalcin, p-carboxy-terminal crosslinking telopeptide of type I collagen (p-CTX), and p-pro-collagen (p-PINP).

### Statistics

Descriptive statistics were used to compare baseline data (including Table 1) and in the listings of all treatment effects, except where a specific test noted in the text. Data were considered non-normally distributed except where noted, and nonparametric tests were used for comparisons. The specific tests used are listed after each comparison in the results section.

**Table 1:** Baseline data

Number of patients	*N* = 24 (#)
Age (years)	45 (Q1 38.5; Q3 57)
Gender (M/F)	7 / 17 (#)
Duration of symptoms prior to referral* (months)	32 (Q1 18; Q3 51)
Duration of antibiotics prior to referral (months)	2.5 (Q1 1; Q3 6)
Follow-up time (months)	23 (Q1 11.5; Q3 42)^+^

Data listed as median with IQR unless where noted

* = First dose of zoledronic acid were given at first visit

+  = 9 patients were still being followed at the end of study

Statistics and general data handling were done using IBM SPSS Statistics v. 23 by IBM Corporation, SIGMAPLOT v. 11.0.0.77 by Systat Software Inc., Microsoft Excel 2000 v. 9.0.2812 by Microsoft Corporation and Apache OpenOffice 4.0.1 by The Apache Software Foundation.

## Results

**Fig. 2:**
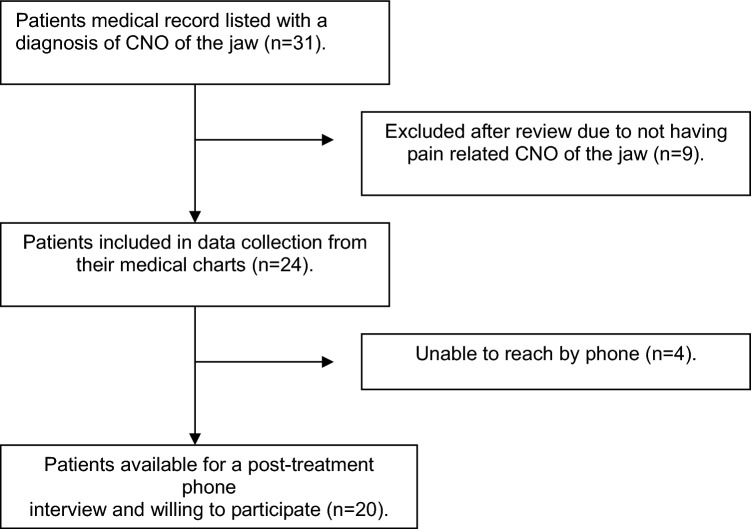
Flowchart of patient inclusion in the study

A total of 31 patients were identified with the referral diagnosis of CNO. After reviewing the medical records individually, 7 patients were excluded due to having another diagnosis than CNO of the jaw. Of these seven, 2 instead had CRMO, 2 were miscoded (they had osteoporosis instead), 1 had osteonecrosis (possibly secondary to Denosumab-treatment), 1 had trigeminal neuralgia, and 1 had pain remission with intensive dental surgery before initiating treatment at our site (diagnosis unknown). Thus, a total of 24 patients were included for further analysis. Patient inclusion in the study can be seen in Fig. 2.

**Fig. 3:**
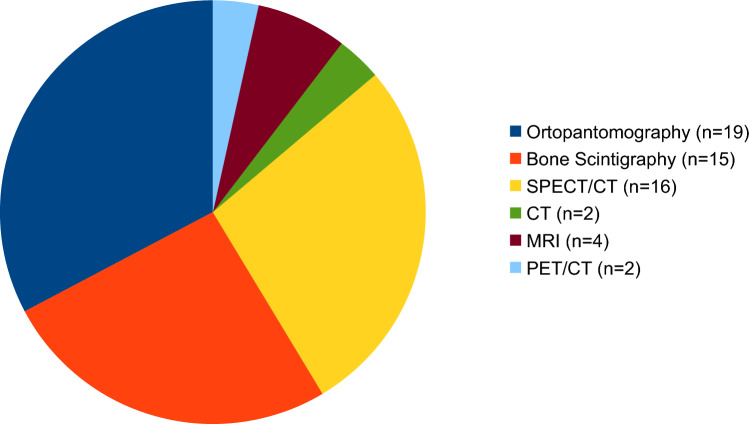
Summary of the diagnostic tests used to confirm the diagnosis of CNO prior to referral

Anthropomorphic data on the 24 included patients are listed in Table 1. Of the 24 patients included, 15 had been discharged from the clinic by the end of the data collection period on March 1, 2023, while 9 were still being followed. A summary of the diagnostic tests used to verify the diagnosis can be seen in Fig. 3.

The average observational period was 33.4 months (median 23.0; Q1 11.5; Q3 42.0).

Patients were initially re-evaluated after the first the first dose of zoledronic acid, either by phone or by physical check-up, after an average of 4.3 months (median 3.5; Q1 1.9; Q3 4.8).

**Fig. 4:**
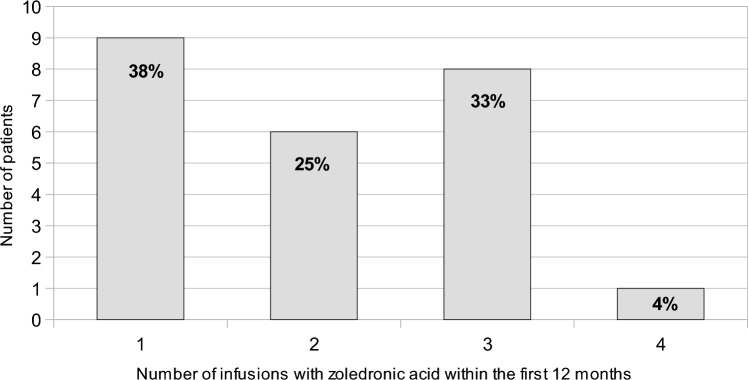
Number of doses of zoledronic acid given within the first 12 months, including initial dose

The average number of infusions of zoledronic acid given was 4.1 (median 3; Q1 2; Q3 5). The total number of doses of zoledronic acid given in the first 12 months can be seen in Fig. 4.

No other treatment than zoledronic acid was given to the patients at the outpatient clinic. As seen in Table 1, patients had received a median of 2.5 months of antibiotical treatment prior to referral. We have no data on concomitant use of painkillers, either as prescription or over-the-counter use.

Blood tests were available for some patients (*n* = 17) from before receiving the first dose of zoledronic acid (baseline) and/or from the end of treatment (final follow-up).

P-CTX deceased significantly from baseline (median 443 ng/L (normal ref. value 125–1477 ng/L) to final follow-up (median 101 ng/L)(rank sum test; *p* < 0.001), while p-alkaline phosphatase did not change from baseline to follow-up (rank sum test; *p* = 0.393).

**Fig. 5:**
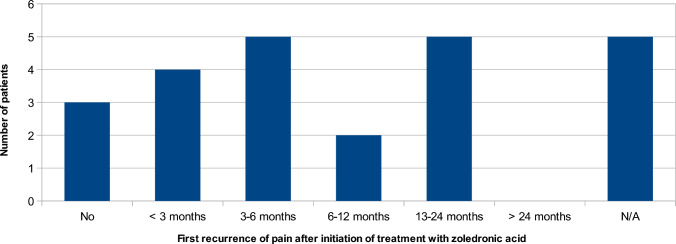
Overview of patients’ 1st recurrence of CNO-related pain. N/A denotes insufficient records data to conclude whether a recurrence had occurred. This was mainly due to insufficient follow-up time (less than 12 months of follow-up)

At least 67% of patients had at least one recurrence of pain after the first documented effect of zoledronic acid, and most experienced the first recurrence within 12 months after being given the initial dose of zoledronic acid (Fig. 5).

**Fig. 6:**
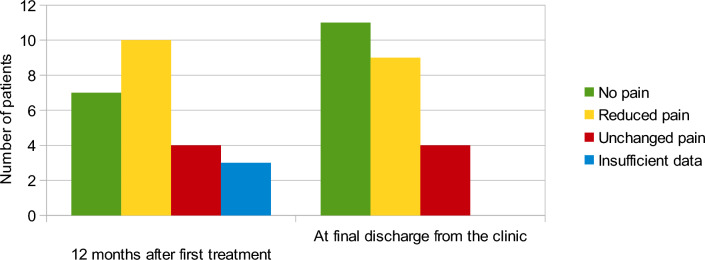
Pain relief after treatment with zoledronic acid

Treatment effect, based on the pain reported by the patients, was recorded after 12 months and at final discharge from the clinic (Fig. 6). Twelve months after initiation of treatment, 71% of the patients reported either no pain or reduced pain.

At final discharge, or end of data collection, 46% of the patients reported no pain and a further 38% had a reduced pain.

Treatment effect was also evaluated by imaging diagnostics in 10 patients (SPECT/CT or bone scintigraphy). Of these 10 patients, 6 had no activity in the jaw area with CNO, 2 had reduced activity, and 2 had unchanged activity. Reduced activity was not uniformly related to pain, as 1 patient with no imaging activity still had unchanged pain, while another patient without any pain still had unchanged activity. Of the rest (*n* = 8), 4 had no imaging activity and reduced pain, 2 had reduced imaging activity and reduced pain, 1 had reduced imaging activity and no pain, and the last patient had unchanged imaging activity and unchanged pain.

A total of 4 patients (16%) had unchanged pain after treatment with zoledronic acid and were thus recorded as such by the end of data collection. They had all been discharged from the clinic due to lack of treatment effect before the date of March 1, 2023. The group consisted of 3 females and 1 male, had received an average of 2 doses of zoledronic acid (range 1–3), and had been followed for an average of 6.8 months (Table 2).

**Table 2:** Comparison of responder versus non-responders

	Responders	Non-responders
Number of patients (#)	20	4
Age (years)	44 (Q1 38.5; Q3 57)	48 (Q1 33.5; Q3 57.5)
Gender (M/F)(#)	6/14	1/3
Duration of symptoms prior to referral (months)	33.5 (Q1 19.5; Q3 51)	22.5 (Q1 11; Q3 49.5)
Number of doses of zoledronic acid (#)	3.5 (Q1 2; Q3 5.5)	2 (Q1 1.5; Q3 2.5)

Data listed as median with IQR unless where noted

There were no significant differences between responders and non-responders regarding gender, age, duration of symptoms prior to treatment, or number of infusions with zoledronic acid given.

### Data from Follow-Up Phone Interview

We were able to reach 20 out of the 24 patients for a follow-up interview by phone.

The average VAS score, as evaluated at the final phone interview, dropped significantly from 7.7 (1.5 SD; median 8; range 5–10) before treatment to 2.5 (2.3 SD; median 2; range 2–7.5) at final discharge or end of data collection (*p* < 0.001, signed rank test).

Patients (*n* = 20) were asked whether they still felt any discomfort from their jaw in the form of intermittent throbbing (*n* = 9), intermittent pain spikes (*n* = 9), and/or reduced function (*n* = 7; 3 of whom had had no effect of the treatment). Patients were also asked if their jaw or face had altered its appearance in their own perception (*n* = 2). Three patients had no symptoms remaining at all.

## Discussion

We have described a population of patients with CNO of the jaw, with a focus on pain relief after treatment with 4 mg intravenous zoledronic acid per dose.

We have shown that zoledronic acid infusion gives a significant pain relief to most patients, with 84% of the patients treated experiencing either reduced or no pain at all after an average of 33.4 months, and a reduction in pain on a VAS by 5.2 points.

Treatment with zoledronic acid for CNO of the jaw is a low-cost intervention to a group of patients who experience significant and often debilitating pain. On average, our population received 4.1 infusions over the course of the treatment period. When used to treat osteoporosis, zoledronic acid is usually given in 3 doses, and thus, in our opinion, our use of zoledronic acid does not expose the patients to an excessive bisphosphonate load compared to normal use. Furthermore, the patients treated suppressed their markers of bone turnover (i.e., P-CTX) to an average value of 101 ng/L, which is just below the reference value and comparable to the values obtained when using zoledronic acid to treat other diseases, such as osteoporosis.

When comparing our findings to those of other authors, who have treated patients with bisphosphonates for CNO/CRMO, we find a similar trend. Both Miettunen et al. [12] and Roderick et al. [16] found clinically significant reduction in pain and focal activity when using Pamidronate to treat juveniles with CRMO or CNO, respectively, while Kuijpers et al. [17] found a similar effect when using Pamidronate to treat patients with specifically jaw-related CNO. Hospach et al. [18] found that Pamidronate appeared to have effect in some juveniles with CRMO where TNF-α inhibitors had not worked previously, although other groups have described positive outcomes using TNF-α inhibitors to treat CNO/CRMO, as summarized by Costa-Reis and Sullivan [10].

Our data also corroborate the findings of several of these groups regarding the importance of extended follow-up time due to the risk of pain recurrence after initial relief.

Thus, our study also shows that some patients who initially responded well to zoledronic acid, might experience one or more pain relapses. Thus, it is important to keep in contact with the patients after initial pain relief for at least 18–24 months.

Furthermore, our follow-up shows that almost all patients will continue to experience some form of discomfort from their jaw even years after treatment, but significantly less than before treatment.

The main limitation of the study is the retrospective observational study design, and as such no fixed schedule was used for the follow-up period and treatment intervals. Thus, it is hard to know if there is a natural progression of pain remission and recurrences.

Furthermore, due to the inconsistencies of blood sampling, it is not possible to get a clear picture of any correlation between pain recurrence and changes in biomarkers.

Finally, the VAS we used to evaluate pain was done retroactively and not while the patient was actually experiencing the pain. Furthermore, in several cases, the interview was done up to 5–6 years after the end of treatment and thus recall bias could influence the values obtained.

Data on use of both over-the-counter and prescription pain relief medication have not been recorded, as these were too sparsely and inconsistently documented in the medical records.

In conclusion, we have found that most patients with CNO of the jaw respond positively to repeated infusions with zoledronic acid with a significantly reduced pain level, although minor symptoms persist for the majority. Due to the heterogeneity of the population and inconsistencies in drug response and frequency of pain recurrence, we are not able to report on a recommended schedule of treatment intervals. More prospective studies of this group of patients are needed, including standardized treatment, intervals of observation, and measures.

### Supplementary Information

Below is the link to the electronic supplementary material.

Supplementary file1 (DOC 13 KB)

## References

[1] Buch K, Thuesen ACB, Brøns C, Schwarz P (2019). Chronic non-bacterial osteomyelitis: a review. Calcif Tissue Int.

[2] Bjrkstén B, Boquist L (1980). Histopathological aspects of chronic recurrent multifocal osteomyelitis. J Bone Joint Surg Br.

[3] Gikas PD, Islam L, Aston W, Tirabosco R, Saifuddin A, Briggs TWR (2009). Nonbacterial osteitis: a clinical, histopathological, and imaging study with a proposal for protocol-based management of patients with this diagnosis. J Orthop Sci Off J Jpn Orthop Assoc.

[4] Jansson A, Renner ED, Ramser J, Mayer A, Haban M, Meindl A (2007). Classification of non-bacterial osteitis: retrospective study of clinical, immunological and genetic aspects in 89 patients. Rheumatol Oxf Engl.

[5] Leclair N, Thörmer G, Sorge I, Ritter L, Schuster V, Hirsch FW (2016). Whole-body diffusion-weighted imaging in chronic recurrent multifocal osteomyelitis in children. PloS one.

[6] Baltensperger M, Grätz K, Bruder E, Lebeda R, Makek M, Eyrich G (2004). Is primary chronic osteomyelitis a uniform disease? proposal of a classification based on a retrospective analysis of patients treated in the past 30 years. J Cranio-Maxillo-fac Surg Off Publ Eur Assoc Cranio-Maxillo-fac Surg.

[7] Catalano-Pons C, Comte A, Wipff J, Quartier P, Faye A, Gendrel D (2008). Clinical outcome in children with chronic recurrent multifocal osteomyelitis. Rheumatol Oxf Engl.

[8] Girschick H, Finetti M, Orlando F, Schalm S, Insalaco A, Ganser G (2018). The multifaceted presentation of chronic recurrent multifocal osteomyelitis: a series of 486 cases from the Eurofever international registry. Rheumatol Oxf Engl.

[9] Obel G, Krogdahl A, Thygesen T, Godballe C (2013). Juvenile mandibular chronic osteomyelitis: 3 cases and a literature review. J Oral Maxillofac Surg Off J Am Assoc Oral Maxillofac Surg.

[10] Costa-Reis P, Sullivan KE (2013). Chronic recurrent multifocal osteomyelitis. J Clin Immunol.

[11] Simm PJ, Allen RC, Zacharin MR (2008). Bisphosphonate treatment in chronic recurrent multifocal osteomyelitis. J Pediatr.

[12] Miettunen PM, Wei X, Kaura D, Reslan WA, Aguirre AN, Kellner JD (2009). Dramatic pain relief and resolution of bone inflammation following pamidronate in 9 pediatric patients with persistent chronic recurrent multifocal osteomyelitis (CRMO). Pediatr Rheumatol Online J.

[13] Camison L, Mai RS, Goldstein JA, Costello BJ, Torok KS, Losee JE (2018). Chronic recurrent multifocal osteomyelitis of the mandible: a diagnostic challenge. Plast Reconstr Surg.

[14] Padwa BL, Dentino K, Robson CD, Woo SB, Kurek K, Resnick CM (2016). Pediatric chronic nonbacterial osteomyelitis of the jaw: clinical, radiographic, and histopathologic features. J Oral Maxillofac Surg Off J Am Assoc Oral Maxillofac Surg.

[15] Wang Y, Yang C, Zhang W, Lu Y, Wei W, Han Z (2017). Monofocal chronic nonbacterial osteomyelitis in the mandible accompanied with mucocutaneous disease. J Craniofac Surg.

[16] Roderick M, Shah R, Finn A, Ramanan AV (2014). Efficacy of pamidronate therapy in children with chronic non-bacterial osteitis: disease activity assessment by whole body magnetic resonance imaging. Rheumatol Oxf Engl.

[17] Kuijpers SCC, de Jong E, Hamdy NAT, van Merkesteyn JPR (2011). Initial results of the treatment of diffuse sclerosing osteomyelitis of the mandible with bisphosphonates. J Cranio-Maxillo-fac Surg Off Publ Eur Assoc Cranio-Maxillo-fac Surg.

[18] Hospach T, Langendoerfer M, von Kalle T, Maier J, Dannecker GE (2010). Spinal involvement in chronic recurrent multifocal osteomyelitis (CRMO) in childhood and effect of pamidronate. Eur J Pediatr.

