# Supplementary material for: Treatment Effect of Zoledronic Acid in Chronic Non-bacterial Osteomyelitis of the Jaw: A Case Series
Source: Calcif Tissue Int. 2023 Nov 22;114(2):129–36. doi: 10.1007/s00223-023-01154-4 (PMC10803718; doi:10.1007/s00223-023-01154-4)
Supplement: Supplementary file 1 — Supplementary file1 (DOC 13 KB) [file 223_2023_1154_MOESM1_ESM.doc]

**Shorthand questionnaire used for the follow-up phone interviews**

1. On a scale from 1-10, how would you describe your pain in the jaw when you were admitted to the endocrinological outpatient clinic?
2. On a scale from 1-10, how would you describe your pain in the jaw presently?
3. Have you had any recurrence of intensified pain episodes since your discharge/last visit?
   - If yes, how long did it last and did it differ from the initial pain sensations?
4. Do you still have any symptoms from the jaw, and if yes, could you please describe them?
   - If yes, how does these symptoms affect your everyday life?
